# Performance of adult-trained artificial intelligence models in paediatric imaging—a scoping review

**DOI:** 10.1007/s00330-026-12354-5

**Published:** 2026-02-12

**Authors:** Lene Bjerke Laborie, Jennifer Lee, Edward Antram, Regina Küfner Lein, Susan Cheng Shelmerdine

**Affiliations:** 1 Mohn Medical Imaging and Visualization Centre, Department of Radiology, Haukeland University Hospital, Bergen, Norway; 2 Department of Clinical Medicine, University of Bergen, Bergen, Norway; 3 Department of Clinical Radiology, Great Ormond Street Hospital for Children, London, UK; 4 Department of Radiology, St George’s Hospital NHS Foundation Trust, London, UK; 5 City St George’s, University of London, London, UK; 6 Medical Library, University of Bergen, Bergen, Norway; 7 UCL Great Ormond Street Institute of Child Health, Great Ormond Street Hospital for Children, London, UK; 8 NIHR Great Ormond Street Hospital Biomedical Research Centre, Bloomsbury, London, UK

**Keywords:** Artificial intelligence, Deep learning, Transfer machine learning, Scoping review, Validation

## Abstract

**Objectives:**

This scoping review aims to evaluate the performance of artificial intelligence (AI) models designed for adults when applied to paediatric imaging datasets without additional adaptations, and to quantify performance degradation across different modalities, use-cases and age groups.

**Materials and methods:**

A literature search was conducted covering 10 years (1/01/2014–23/06/2025) using terms relating to “child”, “adult”, “artificial intelligence”, “radiology” and “validation/performance”. Two reviewers independently extracted data using standardised templates and conducted a narrative analysis.

**Results:**

Of 5642 abstracts, 20 studies met the inclusion criteria. The studies evaluated AI tools across 16 paediatric dataset cohorts ranging from 30 to 7357 subjects. Three datasets were used more than once to evaluate different AI model performance metrics. The tools were applied to radiography (*n* = 7), CT (*n* = 7), MRI (*n* = 2), Dual-energy-x-ray-absorptiometry (DEXA) (*n* = 2) and ultrasound (*n* = 2) across different AI tasks: segmentation (*n* = 9), classification (*n* = 4), detection (*n* = 3), and mixed tasks (*n* = 4). Apart from two studies, all articles reported performance reduction when adult-trained AI tools were applied to paediatric populations. Cohort overlap represents the risk of duplication bias. Detection tasks showed the most severe deterioration, with sensitivity dropping from 68–100% in adults to 26–68% in children for pulmonary nodule detection. For segmentation tasks, Dice score reductions > 0.10 were noted across organs and imaging modalities. Children ≤ 2 years consistently showed the greatest performance deficits across all task types.

**Conclusion:**

AI tools intended for adult use do not perform to the same standard when used in a paediatric population without additional adaptation, particularly for children under 2 years. Careful model evaluation is required before clinical implementation.

**Key Points:**

***Question*** *How do artificial intelligence-based radiology tools designed for adults perform when applied to paediatric imaging without additional adaptation?*

***Findings*** *Adult-trained AI models consistently demonstrated reduced performance in children, particularly in those under 2 years, with detection tasks showing the most severe deterioration*.

***Clinical relevance*** *Healthcare professionals should not assume that adult-trained radiology AI tools intended for adult use can be directly applied to the paediatric population without validation, additional training or fine-tuning, particularly for the youngest age groups.*

**Graphical Abstract:**

**Figure:**
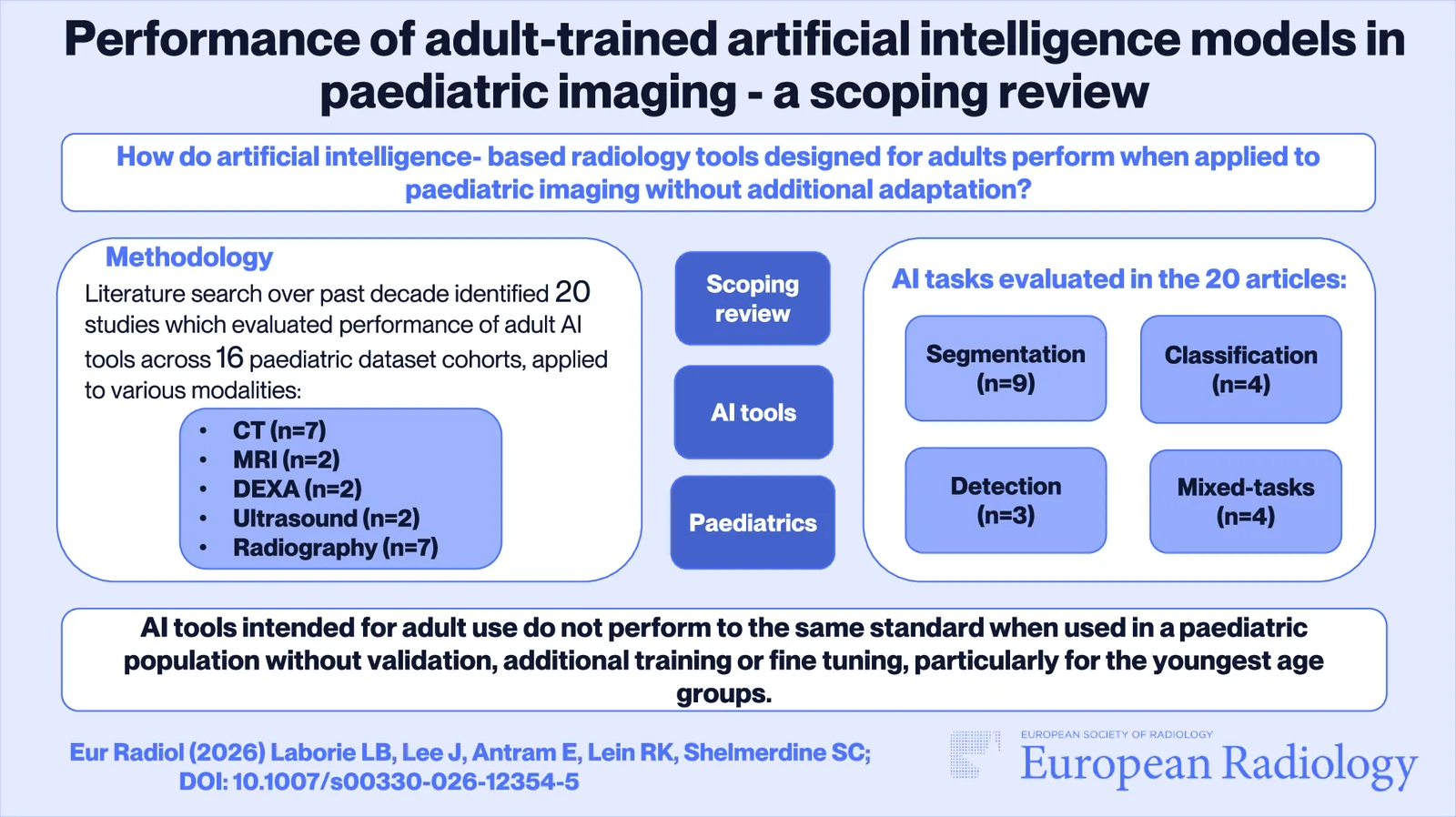

## Introduction

There are now approximately one thousand artificial intelligence-based software as a medical device (AI-SaMD) authorised for clinical use [1]; however, the vast majority are not suitable for paediatric use [2]. This severe lack of paediatric-specific AI tools creates a critical gap in paediatric healthcare where children may be exposed to unvalidated AI tools originally designed for adults. This is also true for paediatric radiology, where only 6 of 196 FDA-approved radiology AI tools [3] were specifically regulated for paediatric use in 2022. The same year, a public warning statement was issued by the American College of Radiologists regarding concerns from the use of adult-trained AI triage tools leading to down-triaging of important paediatric findings and delaying subsequent management [4]. Equally concerning, amongst 149 AI-SaMD labelled for paediatric use in 2024 [5], only 28/149 (18.8%) employed validation datasets that included paediatric patients, while 22/149 (14.8%) were validated exclusively using adult data only, with no information on the remaining 99 (66.4%). A recent review confirmed that most regulated radiology AI medical devices have missing or unclear information regarding the appropriate use in children, with only four (4/213, 2%) of these clearly labelled for paediatric use [6].

This disparity raises fundamental questions about diagnostic accuracy and patient safety when adult-trained algorithms are applied to paediatric populations without appropriate validation [7–10]. Children exhibit distinctive anatomical differences and disease patterns that differ across developmental stages and age groups. These inherent differences may profoundly impact the performance of AI models trained exclusively on adult datasets, potentially compromising clinical decision-making [2]. It is essential to note the importance of rigorous cohort tracking when evaluating published performance metrics of AI models, to avoid bias in outputs of models across homogenous populations. This is particularly important when data and cohorts are scarce.

This review evaluates the performance of adult-trained AI models when applied to paediatric imaging without additional adaptation. We synthesised evidence across different imaging modalities, clinical applications, and age groups to quantify performance degradation patterns and identify specific vulnerabilities. For this scoping review, we aim to include all published evidence, including several publications with slightly different aims emerging from the same cohort. Our analysis aims to establish evidence-based guidance for clinicians regarding the risks of applying unvalidated adult AI tools in paediatric practice and highlight critical gaps requiring paediatric-specific AI development and validation.

## Materials and methods

This review was registered in the Open Science Framework (OSF) Registries (22nd of February 2024) [11] and followed PRISMA extension for scoping reviews (PRISMA-ScR) [12] (Supplementary material—checklist).

### Literature search

We searched MEDLINE (Ovid), EMBASE (Ovid), Web of Science, and the Cochrane Library (Wiley) databases from 1 January 2014 to 23 June 2025. The search strategy, including both subject headings and free text terms, combined artificial intelligence concepts (e.g., “artificial intelligence”, “deep learning”, “machine learning”, “neural network”), paediatric populations (e.g., “paediatric”, “children”, “infant”, “adolescent”), imaging modalities (e.g., “radiology”, “imaging”, “X-ray”, “CT”, “MRI”, “ultrasound”), adult population (e.g., “adults”, “Man” “women”) and “validation/performance”. Additional studies were identified through reference list screening of included articles and relevant review papers (Supplementary material—search strategy).

### Selection criteria

Studies were included if they: (1) evaluated AI models designed for or trained on adult imaging data; (2) reported application of these models to paediatric imaging data (< 21 years old) without additional training or transfer learning; (3) reported quantitative performance metrics; and (4) were published in English.

Studies were included if they evaluated AI models that were originally designed for adult imaging data and applied these unmodified AI models to a paediatric dataset. Studies were also included if they had an element of additional fine-tuning or transfer learning in addition to reporting on, or applying, the unmodified adult-trained AI model.

We excluded studies that: (1) developed AI models specifically for paediatric populations; (2) solely reported outcomes with transfer learning or fine-tuning for paediatric data; (3) were conference abstracts, editorials, or reviews without original data; (4) focused on fetal imaging; or (5) lacked clear paediatric subgroup reporting. We have deliberately included publications emerging from overlapping external validation cohorts, as the publications have differences with respect to aims and/or models evaluated and outputs discussed, and as this scoping review aims to synthesise all available evidence. Overlapping publications are clearly marked with asterisks in all the tables for transparency.

### Data extraction and quality assessment

At present, there are no AI-specific tools for evaluating the risk of bias of articles, even though these are pending publication [13, 14]. Therefore, we assessed methodological quality and risk of bias using a modified QUADAS-2 framework [15] supplemented with AI-specific considerations from the CLAIM guideline [16], which has been used in prior AI-related systematic reviews in the past [17, 18].

Each study was evaluated across three domains: patient selection, index text (i.e., AI tool), and reference standard, with both risk of bias and applicability concerns rated as low, high, or unclear. Specifically, these were evaluated as follows:

Risk of bias:

- Patient Selection: consideration regarding appropriate patient selection for the intended task, collating a balanced dataset, suitable data sources, and unreasonable/extensive exclusion criteria.
- Index test (AI): consideration of measures of significance and uncertainty of the AI tool, transparency of methods and training of the tool/description of the architecture.
- Reference Standard: sufficient detail to allow replication of ground truth/reference standard, blinding to clinical details, rigorous evaluation to know that the disease/finding is genuinely present.

Applicability Concerns:

- Patient Selection: how applicable/useful the algorithm is for paediatric usage, given the population being tested in the study.
- Index test (AI): suitable information on validation or testing of the algorithm on external data.
- Reference Standard: appropriateness for clinical practice, as applied to children.

Since all AI models being evaluated were not specifically designed or validated for children, the risk of bias and applicability concerns for the index text were expected to be high for all articles a priori, and we did not plan to exclude any articles based on these risks. Two reviewers independently extracted bias/applicability and article data using a standardised data extraction template. Extracted information from each article included study characteristics (author, year, country), AI model details (architecture, training data), paediatric population characteristics (age range, sample size, pathology), and performance metrics.

### Data synthesis and analysis

Due to the heterogeneity in AI applications and metrics, we conducted a narrative synthesis rather than a meta-analysis, organising findings by AI task application. Given the scoping nature of this review, formal qualitative assessment tools were not applied, though study methodology and design were considered when interpreting the results.

## Results

**Fig. 1:**
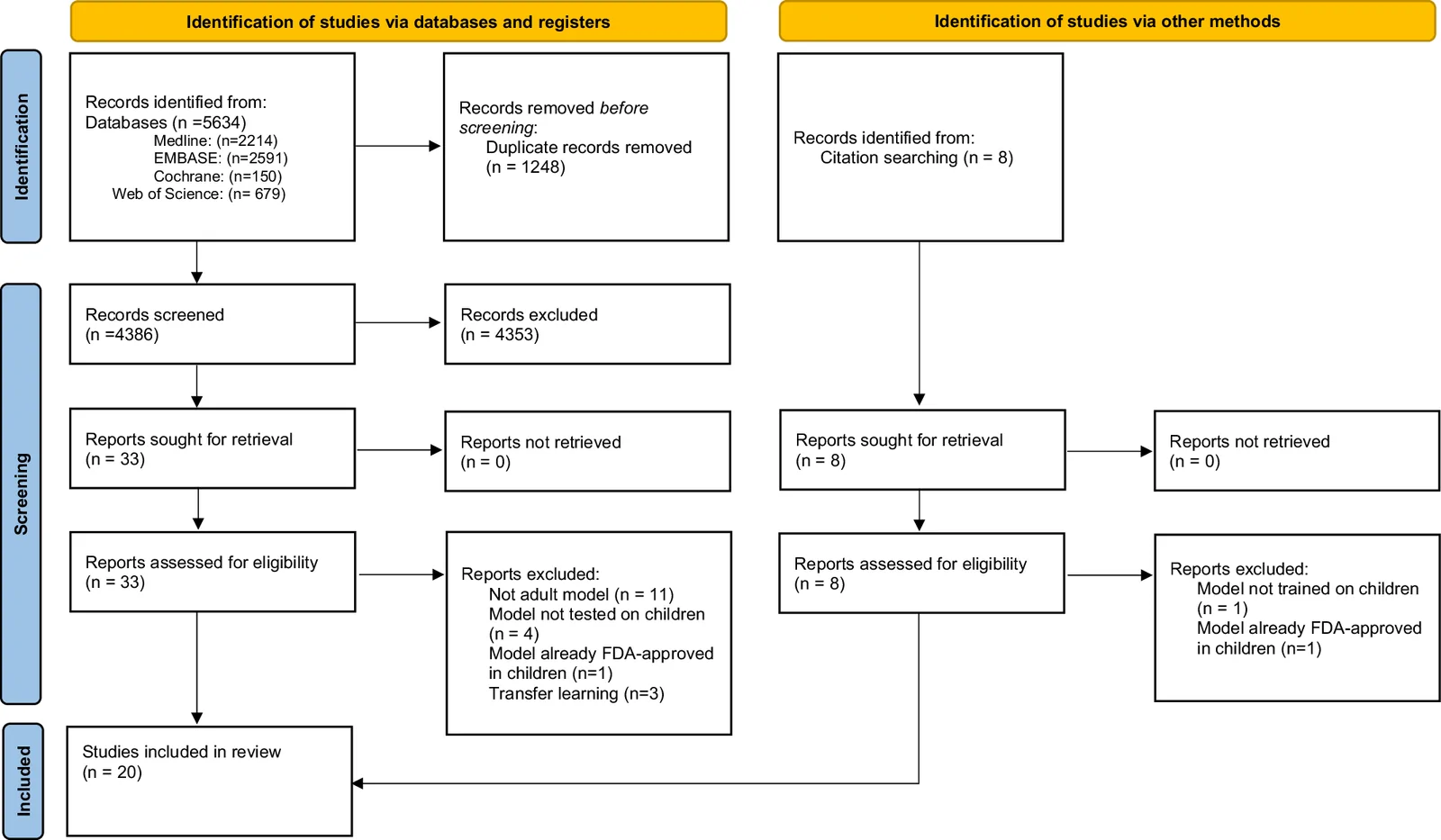
Prisma flowchart for scoping review, with inclusion of 20 studies, reporting on 16 paediatric dataset cohorts. Prisma 2020 flowchart. Source: Page et al, BMJ 2021;372:n71. doi: 10.1136/bmj.n71. This work is licensed under CC BY 4.0. To view a copy of this license, visit https://creativecommons.org/licenses/by/4.0/

Our material consisted of 5642 abstracts from literature searches and citation searches, of which 20 studies [19–38] met the eligibility criteria for final inclusion (Fig. 1: Prisma flowchart).

### Risk of bias and applicability

The risk of bias and applicability for the studies were summarised (Suppl. Table 1). Across the 20 studies, the most frequent source of bias was the index test due to the AI tool being developed on adult datasets, leading to risk of bias and applicability. Patient selection was also at high risk in most studies, due to the use of single-centre, non-consecutive, or highly selective datasets with limited applicability and generalisability due to narrow age bands, rare diseases or unclear description of the paediatric dataset. The reference standard was generally appropriate for most studies that used consensus expert reads, expertly trained manual delineation for segmentation tasks, or, in some cases, histopathology. In a few studies, where a single rater without blinding was used or labels derived from dataset annotations without explicit clarification as to how these were derived, the applicability and bias were rated as high or unclear.

We acknowledge that three paediatric datasets for AI model validation were used more than once (see Tables 1–3), although the research questions and model evaluation applied to these datasets differed. Nonetheless, given that there may be some bias in how the patients in the datasets were selected and a lack of transparency in their ethnicity and socioeconomic status, one must be mindful that the AI model performance may not be generalisable to all children from all backgrounds.

**Table 1:** Notable inclusion and exclusion criteria for the paediatric (test) dataset, including ground-truth labelling (*n* = 20 articles)

Author, year	Modality	Notable paediatric data included in the test dataset	Notable exclusion criteria	Readers/ground-truth labelling
Candemir, 2015	Radiography (Chest)	Paediatric patients across different developmental stages (infancy to adolescence) to study lung shape changes. The study specifically focused on tuberculosis screening applications.	No specific exclusions mentioned for paediatric patients.	No specific mention of who performed the manual delineations for paediatric cases.
Alqahtani, 2017*	Radiography (Lateral thoracolumbar spine, T4-L4)	199 (80%) had suspected reduction in BMD, including children with osteogenesis imperfecta, inflammatory bowel disease, rheumatologic conditions, cystic fibrosis and coeliac disease. The remaining 51 (20%) were recruited from the spine clinic	Images where observers were unable to identify T12 and/or L1 (e.g., due to excessive coning) were not scored	Previous consensus arrived at by three paediatric radiologists using a simplified algorithm-based qualitative (ABQ) technique served as the reference standard
Alqahtani, 2019*	Radiography (Lateral spine) and DEXA (Thoracolumbar spine)	80% had suspected reduced BMD (e.g., osteogenesis imperfecta, inflammatory bowel disease, rheumatological conditions, cystic fibrosis) attending metabolic bone clinic. 20% were attending spine clinics for suspected scoliosis	Not stated	Previously established consensus arrived at by three experienced paediatric musculoskeletal radiologists using a simplified algorithm-based qualitative (sABQ) scoring system
Alqahtani, 2020*	Radiography (Lateral spine) and DEXA (Thoracolumbar spine)	380 (90%) had osteogenesis imperfecta, 12 (3%) Duchenne muscular dystrophy, 8 (2%) polyostotic fibrous dysplasia, and 20 (5%) other conditions, including anorexia nervosa, diabetes mellitus, juvenile dermatomyositis and coeliac disease.	4% of vertebrae were not evaluable due to poor visualisation or poor image quality, including movement artefact. The majority of unevaluable vertebrae were located in the upper thoracic spine.	An experienced paediatric radiologist with 20 years’ experience in paediatric radiology using Genant’s semiquantitative scoring system served as the gold standard.
Bermudez, 2020	MRI (Brain, T1-w, 3 T field strength)	Paediatric subjects with MRI T1-weighted brain MRI, 3 T field strength	NS	Initial automatic segmentation from multi-atlas segmentation, then manually corrected by expert rater in paediatric imaging
Shin, 2022**	Radiography (Chest)	All paediatric pts ≤ 18 with PA/AP chest radiographs in time frame	Lateral or decubitus chest radiographs excluded	A board-certified paediatric radiologist with 11 years of experience evaluated each chest radiograph. Patient information, including age, clinical data, and results from other available imaging studies, was provided to minimise false decisions.
Hardie, 2023	CT (Chest)	Children with a history of known or suspected malignancy undergoing chest CT for staging and follow-up. All nodules 3–30 mm were included for analysis.	Patients 18 years and older were excluded. Reviewers were instructed to report lung nodules only and not findings consistent with infection or lung masses measuring more than 3 cm.	Three board-certified fellowship-trained paediatric radiologists initially identified lung nodules. For the current study, secondary review was performed by one of two board-certified fellowship-trained paediatric radiologists (with 2 and 11 years of post-training experience) who annotated nodule coordinates and performed segmentation.
Morcos, 2023	Radiography (Chest)	Frontal view CXRs of paediatric patients aged 1–5 years in JPEG format. Cases labelled as normal, bacterial pneumonia, or viral pneumonia. All chest radiographs had been screened for quality control.	Images were screened for quality control before labelling	Labelled by two expert physicians as normal, bacterial pneumonia, or viral pneumonia
Rajaraman, 2023	Radiography (Chest)	Paediatric CXRs from various age groups divided into three developmental stages based on lung shape changes from infancy to adulthood. Includes both normal and abnormal lungs	For adult datasets, 43 CXRs from Shenzhen were excluded that erroneously included heart regions in the GT lung masks. No specific exclusions mentioned for paediatric dataset	Not explicitly stated for the paediatric dataset
Salman, 2023***	CT (Chest and TAP)	Children 12–18 years old with chest CT examination	Exams in children < 12 years old were not eligible for inclusion. No studies were excluded for low image quality or artefacts	Two board-certified paediatric radiologists (13 and 15 years of post-paediatric radiology fellowship experience) re-interpreted CT images independently and then reached consensus. They were blinded from clinical history, original radiologist reports, comparison CT scans, pathology reports, and the patient’s electronic medical record.
Salman, 2023***	CT (Chest and TAP)	Children 12–18 years old with chest CT examination	< 12 years excluded	Two board-certified paediatric radiologists (13 and 15 years of post-paediatric radiology fellowship experience) re-interpreted CT images independently and then reached consensus. They were blinded from clinical history, original radiologist reports, comparison CT scans, pathology reports, and the patient’s electronic medical record.
Yang, 2023	Ultrasound (Thyroid)	Only pathologically confirmed thyroid nodules, which included both papillary thyroid carcinomas and benign nodular goitres. Patients with external ultrasound examinations were included, where available	Nodules without pathological confirmation, no available ultrasound imaging for review, non-diagnostic imaging, diagnosis of MEN	Histopathologically diagnosis (from surgical specimens or biopsy) served as the ground truth. Comparison of model performance for overall impression was also compared with the performance of three radiologist readers (seniority level not stated) and ACR TI-RADS classification scores.
Chatterjee, 2024	CT (Abdo/Pelvis)	Paediatric CT dataset comprised scans from patients with a variety of indications, including trauma, cancer staging, and congenital abnormalities. 11 organs were included: right and left adrenal glands, bladder, duodenum, gallbladder, right and left kidneys, liver, pancreas, spleen, and stomach.	Two organs were excluded: prostate (TotalSegmentator version didn’t segment prostate) and oesophagus (inconsistent anatomical borders between datasets). Images with poor visualisation or quality issues.	Expert medical analysts using ProKnow contouring software with annotations reviewed by a board-certified radiation oncologist
Chen, 2024	CT (Head and Neck)	Paediatric patients with tumours in the central nervous system (CNS) and head & neck region. Patients were treated in various orientations (eight different patient positions). Some patients had immobilisation equipment, including bite positioners or intubation equipment.	No specific exclusions mentioned for paediatric patients. General exclusions included cases where the model failed to localise organs at risk.	Manual segmentations were made by radiation oncologists and radiation technologists using the RayStation treatment planning system. No specific mention of who labelled the paediatric cases specifically.
Kumar, 2024	CT (Chest/Abdomen)	Seven pelvic/thoracic organs (bladder, heart, liver, left kidney, right kidney, pancreas, and spleen). CT scans from chest/abdomen/pelvis and abdomen/pelvis regions. All scans had human expert ground-truth contours.	Not stated	Expert physicians made the segmentation contours. For TCIA data, expert organ contours were made by expert physicians. For clinical data, expert contours were made during treatment planning and approved by physicians.
Rollan-Martinez-Herrera, 2024	Radiography (Chest)	Among the 5856 paediatric images: 4273 cases diagnosed with pneumonia (1493 viral pneumonia, 2780 bacterial pneumonia), and 1583 healthy subjects with no signs of pneumonia.	Images with two or more external devices (cables, leads, tubes, pacemakers) over the lung field were removed. Images with extreme angular rotations, positioning, or incorrect pulmonary fields (image truncation) were omitted.	Three expert physicians screened the paediatric images for quality control and diagnosis. For the adult training dataset, five trained readers reviewed and classified images, with label consensus reached
Lhermitte, 2024	MRI (Brain, T1-w)	Derived from the CAP dataset, originally scanned at 4 sites before and after motor therapy. Children with stroke lesions resulting in cerebral palsy were included. Lesions ranged in size from 2.5 to 172 mL in volume	Images with excessive artefacts were excluded	Medical experts (not otherwise specified) manually segmented images using ITK-SNAP. Segmentations for the follow-up lesions (session 2) were derived via registration of session 1 masks and visually checked for accuracy.
Shin, 2024**	Radiography (Chest)	All paediatric pts ≤ 18 with PA/AP chest radiographs in time frame	Radiographs with artefacts caused by imaging errors (caregivers’ hands, external objects overlapping the thoracic region)	A board-certified paediatric radiologist with 12 years of experience evaluated each chest radiograph.
Ha, 2025	Ultrasound (Thyroid)	150 consecutive paediatric patients (aged < 18 years) with thyroid nodules ≥ 5.0 mm who underwent US and FNA at two institutions. Patients with definitive benign or malignant diagnoses based on surgical specimens or biopsies were included. Malignant nodules included papillary thyroid carcinomas (including follicular variants), follicular carcinomas, medullary carcinomas, and poorly differentiated thyroid carcinoma. In total, 156 thyroid nodules were assessed according to the relevant adult guidelines (American College of Radiology (ACR)- and Korean (K)-TIRADS)	Lack of a definitive diagnosis (Bethesda category I, III–V) on biopsy without surgical confirmation (*n* = 18) or suboptimal image quality (*n* = 4)	Paediatric thyroid experts (paediatricians, radiologists, endocrinologists, or surgeons) reviewed the patient’s clinical presentation and US images before recommending FNA. Definitive benign or malignant diagnosis was based on surgical specimens or biopsies (Bethesda category II or VI).
Thibodeau-Antonacci, 2025	CT (18 organs at risk, OARs, in the context of craniospinal irradiation)	CT scans from 43 (27 + 16) paediatric patients were included for testing. All scans underwent atlas-based segmentation of OARs (*n* = 18) followed by manual correction.	Excluding scans without images covering from above the head to the thigh and a patient with severe scoliosis	All images underwent manual delineation of 18 OARs

The dataset study period denotes the time frame over which the paediatric test dataset was acquired, unless explicitly stated otherwise. All datasets were retrospective in nature

There were three paediatric test datasets that were used more than once across different publications. Where this is the case (i.e., more than one study using the same test dataset), we have denoted this with asterisks. In summary, the same test dataset was used across Alqahtani 2017, 2019 and 2020 (*); another was used for Shin 2022 and Shin 2024 (**), and also Salman 2023 (***)—two publications for the same year using the same dataset

*CXR* chest radiograph, *NS* not stated, *DEXA* dual-energy X-ray absorptiometry, *CT* computed tomography

**Table 2:** Study aims and performance differences between adults and paediatric datasets for segmentation-based tasks (*n* = 9 articles), classification-based tasks (*n* = 4 articles), detection-based tasks (*n* = 3 articles), and mixed-task-based models (detection and classification) (*n* = 4 articles)

Author, year	Disease/task	Study aims	Baseline performance in adults	Main takeaway/conclusions
Candemir, 2015	Lung boundary detection. Segmentation.	To enhance an existing adult chest X-ray screening system to include paediatric CXR images by quantifying changes in lung shape from infancy to adulthood and developing new lung models suitable for paediatric developmental stages.	The adult lung segmentation model achieved a peak DICE score of 95.4% ± 0.015 in adult radiographs.	Paediatric lung shapes are different from adults, especially in infants. Optimising the originally trained adult model using registration-based techniques to better recognise children’s lung shapes produced more accurate segmentation models. By adapting the adult model, DICE scores improved from 36.8% to 86% (< 2 years old); from 83.2% to 92.5% (2–11 years old) and 93.8% to 94.5% (for 11–18 years old). The youngest age group saw the greatest improvement with model optimisation, the oldest children’s age group only demonstrated marginal benefits as their lung morphology was similar to adult anatomy.
Alqahtani, 2020*	Vertebral fractures. Segmentation.	Determine the diagnostic accuracy and inter-/intra-observer agreement of morphometric vertebral fracture analysis (MXA) using a 33-point software (designed for adults) on children with conditions predisposing to vertebral fractures, compared to the reference standard of visual semiquantitative method by experienced paediatric radiologist.	Original adult validation data not presented in this work, but has been reported that adult morphometric vertebral fracture analysis systems typically achieve sensitivity of 71–100% and specificity of 18–94%	In children, the morphometric fracture analysis system achieved an overall sensitivity of 80%, specificity 90%, false-positive rate 10%, false-negative rate 20%. This was poorest when evaluating the mild vertebral fractures in children, with a sensitivity of only 46% (but high specificity of 92%, false-positive rate 8% and false-negative rate 54%). The reason quoted was that it was difficult for the software to distinguish mild wedging (physiological) from actual mild fractures (many normal variants inappropriately labelled).
Bermudez, 2020	Whole brain segmentation. Segmentation.	To improve the generalisability of the adult-trained whole brain segmentation algorithm (SLANT) to paediatric populations and contrast-enhanced imaging using augmented transfer learning.	Not specifically stated in this article, but reference to prior reports by the model developers showing that multi-atlas segmentation achieved DSC of 0.760 ± 0.012 in an adult cohort. The authors did not report the performance of the SLANT on their withheld dataset of the adult population.	The following performance metrics were noted when the different original SLANT (adult model) and re-trained SLANT models were applied to the withheld paediatric dataset: Baseline SLANT (intended for adult population): DSC 0.82 Paediatric-only transfer learning (pSLANT): DSC 0.90 Mixed adult + paediatric transfer learning (mpSLANT): DSC 0.89 The re-trained SLANT model performance fell when applied to the adult dataset afterwards: Paediatric-only transfer learning (pSLANT): from 0.715 to 0.698 Mixed adult+ paediatric transfer learning (mpSLANT): from 0.715 to 0.704 Augmented transfer learning (mixing original adult data with new paediatric data) preserves performance on adults while significantly improving paediatric performance. Pure transfer learning on paediatric data alone degrades adult performance.
Rajaraman, 2023	Lung boundary detection. Segmentation.	To analyse the generalisability of deep adult lung segmentation models to the paediatric population and improve performance through a stage-wise, systematic approach consisting of CXR modality-specific weight initialisations, stacked ensembles, and an ensemble of stacked ensembles	Not explicitly stated—the paper focuses on cross-domain generalisation from adult to paediatric populations	For the models trained entirely on adults then applied to the full paediatric dataset, the best performing models across the three age groups was the Inception-V3-UNet (DICE: 0.92 to 0.97), with the model performing better for the older P3 subgroup. When this Inception-V3-UNet model was trained using the paediatric dataset in isolation (compared to other combinations of using adult datasets) this made a big difference when tested on outcomes for the P1 age group (0.93 versus 0.64–0.88 DICE scores when using adult data for training), but not for the P2 or P3 age groups where training the model on adult data either matched or outperformed the same model performance when using dedicated paediatric data for training (for P2 the Inception V3-UNet achieved DICE of 0.95 when trained on paediatric data (identical for adult-trained model) and for P3 it achieved a DICE of 0.97 when trained on paediatric data versus 0.98 when trained on adult data). Therefore, adult lung segmentation models show significant domain shift when applied to paediatric populations, particularly for the youngest age group (P1: 1 day to < 24 months).
Chatterjee, 2024	Organ segmentation (not disease specific). Segmentation.	To evaluate the generalisability of an adult-trained deep learning CT scan organ segmentation model (TotalSegmentator) to paediatric CT scans, and to evaluate the utility of two deep learning optimisation techniques to improve paediatric segmentation performance. One of these involving training a completely new model using only paediatric data, and a second technique fine-tuning a pretrained adult model on paediatric data.	TotalSegmentator achieved a mean Dice score of 0.81 (0.80–0.81, 95% CI) on the adult AMOS dataset (*n* = 300).	In children, the mean DICE score was 0.73 (0.72–0.74, 95% CI) compared to 0.81 in adults (p < 0.001), with the worst performance drops in children seen in the adrenal glands (40–49% performance drop) and duodenum (30% performance drop). The youngest age groups (0–4 years old) also demonstrated the greatest performance drop, notably for left adrenal and pancreas. Paediatric-optimised models significantly improved performance regardless of whether this was using only paediatric data for training or adult training data with fine-tuning using paediatric data. There was no statistical difference between their performances, but all improvements in DICE scores across all organs showed a significant improvement when compared to the adult data trained only model. DICE scores for the adrenal glands showed a 47–66% improvement for the paediatric optimised models (compared to the standard adult model), the performance in the duodenum improved by 79%. All other organs demonstrated DICE scores of > 0.8 with the paediatric optimised models.
Chen, 2024	Head and neck and central nervous system tumours. Segmentation.	To assess the performance of a commercial auto-contouring model for head and neck (H&N) patients in eight different orientations from geometric, dose/volume and qualitative perspectives, focusing on validation and implementation for routine clinical use in particle therapy with fixed beam lines.	The study evaluated performance in the entire study population (adults and children) without differentiating the metrics (except in the discussion to state there was no significant difference between the two groups). However, with non-standard positioning, some organs had a DSC of 0.0–0.1 (rotated/prone positions)	No significant geometric differences were found between paediatric and adult autocontours for any of the analysed organs at risk. The model performed adequately for head-first-supine straight and hyperextended orientations in both adult and paediatric patients, with 13/16 organs at risk being suitable for clinical use. This indicates that the adult-trained model can adapt to different proportions found in paediatric head and neck regions without significant performance degradation, so long as standard positioning is followed. Nevertheless, performance dramatically deteriorated in non-standard patient positions for adults and children, with some organs showing DSC as low as 0.0–0.1 in rotated and prone positions.
Kumar, 2024	Organ segmentation (not disease specific). Segmentation.	To evaluate the performance of AI-based auto-segmentation models trained on adult CT data when applied to paediatric datasets, explore the improvement in performance gained by including paediatric training data, and examine their ability to accurately segment CT data acquired from different scanners.	The version of the AI model trained only on adult data achieved strong performance on adult test data with mean DSC ranging from 0.8–0.95 across all organs, while the adult & paediatric-trained model performed comparably (0.84 to 0.95 DSC) without statistical difference when applied to the adult dataset.	The adult-only trained model performed poorly in children, especially for the younger age group (DSC 0.07 to 0.65 across different organs for the 0–2 age group, compared to 0.80 to 0.92 in adult data). Using a model trained only on paediatric data achieved a DSC 0.78–0.97 in all organs in every age group. Using a model that combines paediatric and adult training data was not statistically different to the paediatric-only trained model, and achieved a DSC of 0.79–0.97 across all organs and age groups. For optimal segmentation in a paediatric population, it is important to include paediatric data in the training of segmentation models.
Lhermitte, 2024	Stroke lesions. Segmentation.	To evaluate whether adult-trained stroke lesion segmentation models can generalise to paediatric brain MRIs. Secondary aim was to identify the deep learning architecture with the best performance for this segmentation task	Best performance acquired on training the models on a subset of the ATLAS dataset, yielding DSC scores of 0.54 (U-Net), 0.68 (Res-U-Net) and 0.79 (nnU-Net)	In the paediatric cohort, the DSC scores were 0.40 (U-Net), 0.48 (Res-U-Net) and 0.57 (nnU-Net), respectively, without fine-tuning. Adult-trained models showed reduced performance on paediatric data, with lower Dice similarity coefficients. The highest performing model architecture, however, was the nnU-Net for paediatric stroke lesion detection.
Thibodeau-Antonacci, 2025	18 Organs at risk in the context of paediatric craniospinal irradiation (CSI). Segmentation.	Evaluate auto-segmentation approaches, including testing of the adult tool LimbusAI, developed specifically for radiotherapy purposes, in a paediatric population.	Not explicitly stated	Commercial tool LimbusAI could not adapt well to paediatric anatomy, in particular for the oesophagus (distal oesophagus was often not segmented), and for the kidneys, where contours were frequently truncated
Morcos, 2023	Pneumonia. Classification.	To investigate the performance of an adult-trained algorithm in detecting pneumonia in a paediatric population, to explore the viability of leveraging adult-trained algorithms to accelerate paediatric AI research	Baseline performance of this model for pneumonia detection on adult radiographs was described as “acceptable” with AUC scores ranging from 0.7 to 1.0 depending on the dataset.	The AI model achieved an accuracy of 76.54% accuracy, sensitivity of 79.83%, and specificity of 67.66%, with F1 = 0.83 and PPV 86.9% and NPV 55.4% and AUC of 0.74 (overall and for bacterial and viral pneumonia) in children. Viral pneumonia had a lower PPV (69.95) and F1 = 0.74. Although the AI model was predominantly trained on adult data, there was some paediatric imaging in the training dataset (< 5%). When compared to performance in adult populations, the AUC in the paediatric dataset is at the lower end of the range of baseline AUC scored in adult pneumonia detection. The authors compare their model performance to those of other references which evaluate AI models trained on paediatric data (and applied to the same paediatric dataset at they report in this study) and found the performance of these paediatric-specific tools ranged in AUC scores between 0.91 to 0.99.
Yang, 2023	Thyroid nodules. Classification.	To compare the diagnostic performance of radiologists’ overall impression, the American College of Radiology Thyroid Imaging Reporting and Data System (ACR TI-RADS) score, and a deep learning algorithm (trained on adults) for differentiating benign and malignant thyroid nodules on ultrasound in children.	Prior performance in 99 thyroid nodules in adults, referenced in the article where the AI model was developed, reported 87% sensitivity, 52% specificity (similar to expert radiologists evaluated). The cohort here had a mean age of 53.2 years (range 19–82 years)	Although the AI model evaluating the paediatric dataset had a higher sensitivity than radiologists evaluating the same paediatric thyroid nodules (87.5% versus mean 58.3% sensitivity), it had a lower specificity (36.1% versus mean 79.9%). Compared to performance in an adult population (sensitivity 87%, specificity 52%), this tool had a similar sensitivity but worse specificity when applied to children.
Rollan-Martinez-Herrera, 2024	Pneumonia. Classification.	To investigate the feasibility of applying CNNs trained on adult chest X-ray images to paediatric populations for pneumonia classification	On the adult dataset, the CNN achieved excellent performance: AUC of 0.95 (95% CI: 0.94–0.95), with F1 score of 0.82, precision of 0.87, accuracy of 0.82, and recall of 0.82.	The adult-trained pneumonia CNN achieved an AUC of 0.82 (95% CI: 0.81–0.83) on paediatric data compared to 0.95 on adult data—a reduction of 0.13 in AUC but still clinically useful performance.
Ha, 2025	Thyroid nodules. Classification.	The AI-Thyroid deep learning (DL) model, originally trained on adult data, was tested on paediatric nodules on US images. The model’s performance was compared with radiologist interpretations using the adult-based Thyroid Imaging Reporting and Data System (TIRADS).	Previous studies involving adult populations have reported AUROC values ranging from 0.922 to 0.938.	This study evaluated the diagnostic performance of AI-Thyroid on two paediatric datasets. AI Thyroid yielded AUROC values of 0.913–0.929 without significant differences across different image planes (axial vs longitudinal planes) and between the two cohorts, despite variations in malignancy prevalence (41.0% vs 17.8%) and geographic settings (South Korea vs the United States). These findings suggest that AI-Thyroid, previously validated in adults using large-scale multicenter data, may also be applicable to paediatric populations.
Hardie, 2023	Pulmonary nodules.Detection.	To evaluate the diagnostic performance of traditional and deep learning CAD systems trained with adult data for the detection of lung nodules on chest CT scans in children, and to compare their ability to generalise to children versus to other adults.	FlyerScan: 83.9% sensitivity at 2 false positives per scan on adult dataset; MONAI: 95.5% sensitivity at 2 false positives per scan on the same adult dataset	FlyerScan sensitivity dropped from 83.9% in adults to 68.4% in children (absolute decrease of 15.5%). MONAI sensitivity dropped from 95.5% in adults to 53.1% in children (absolute decrease of 42.4%). The performance difference was attributed to smaller nodule sizes in paediatric patients (mean 5.4 ± 3.1 mm in children vs 11.0 ± 6.2 mm in adults).
Salman, 2023***	Pulmonary nodules. Detection.	To test the performance of a commercially available adult pulmonary nodule detection artificial intelligence (AI) tool in paediatric CT chests, evaluating the algorithm’s performance at different slice thicknesses (3 mm and 1 mm).	In adults, reported sensitivities range from 68% to 100% with a specificity of 70% in some studies.	The adult Lung CAD showed very low sensitivity in paediatric patients (39% at 1 mm, 26% at 3 mm) when considering all nodules. Performance improved significantly when smaller nodules were excluded based on software constraints—sensitivity increased to 68% at 1 mm and 49% at 3 mm slice thickness. The AI performed better at a thinner slice thickness (1 mm vs 3 mm). However, even with optimal conditions, performance remained substantially lower than reported adult performance, indicating the adult-trained AI is not suitable for paediatric patients without significant modifications.
Salman, 2023***	Pulmonary nodules. Detection.	To determine the diagnostic performance of commercially available Computer-Aided Detection (CAD) for pulmonary nodules in paediatric patients at simulated lower radiation doses, comparing performance at standard dose versus 75%, 50%, and 25% simulated dose reductions.	Expected adult performance metrics not explicitly stated in the article.	The Lung CAD showed extremely low sensitivity (24–27%) at all simulated radiation doses for detecting pulmonary nodules in this paediatric population. However, radiation dose reduction down to 25% of the original dose did not significantly compromise the already poor AI performance—there was no statistically significant difference between dose levels (*p*-values of 1.0, 1.0, and 0.7 for 75%, 50%, and 25% vs standard dose). While the adult-trained CAD performed poorly in children, the radiation dose can be reduced without further degrading its limited performance
Alqahtani, 2017*	Vertebral fractures. Mixed.	To assess the observer reliability and diagnostic accuracy of the semi-automated 6-point technique (developed for VF diagnosis in adults) when applied to children and adolescents using SpineAnalyzer software	The performance in adults is quoted as high with reported sensitivity of up to 94%, specificity up to 100% and reader agreement of 0.96–0.97 (excellent).	Overall 6-point technique sensitivity was poor at 18% (95% CI: 14–22), specificity of 97% (95% CI: 97–98). Not accurate/reliable for vertebral fracture diagnosis in children, with only moderate agreement (kappa up to 0.47 (fair-moderate)). T4 vertebral body was the least readable vertebral level across the imaging studies, with the upper thoracic spine (T4-6) having consistently poor visibility and fractures here more likely to be missed (and more likely to occur in children). Although the software appears useful in adults, due to its low inter- and intra-observer reliability and sensitivity, the tool was deemed inappropriate for paediatric use.
Alqahtani, 2019*	Vertebral fractures. Mixed.	To assess whether the diagnostic accuracy of morphometric vertebral fracture (VF) diagnosis in children can be improved using AVERT™ (33-point program) compared with SpineAnalyzer™ (6-point program)	Previous adult studies show that 6-point technique programs have very high sensitivity and specificity, reaching 98% and 99% respectively, with excellent inter-observer agreement of 99% and kappa ranging from 0.86 to 0.97	Overall sensitivity ranged from 26–31% for SpineAnalyzer™ and 36–41% for AVERT™ in children. Both had low accuracy and poor reliability (kappa up to 0.5). Neither software programme is satisfactorily reliable for vertebral fracture diagnosis in children
Shin, 2022**	Eight detectable lesions on radiography: nodules, consolidation, fibrosis, atelectasis, cardiomegaly, pleural effusion, pneumothorax, pneumoperitoneum. Mixed.	To evaluate whether AI-based lesion detection software that was developed and approved for adult chest radiographs could be used for paediatric chest radiographs, and to identify specific age groups for which the software needs further validation before clinical application.	The vendor reported diagnostic accuracy is 97–99% for adults.	AI-based software developed for adults showed significantly worse performance in very young children, particularly those ≤ 2 years old. When cardiomegaly was excluded and children ≤ 2 years old were excluded, the AI achieved 96.9% accuracy comparable to adult performance (97–99%). However, for the full paediatric population, including all lesions, accuracy was only 87.5%. Age was a significant factor for incorrect results (odds ratio 0.821), with 81.5% of incorrect diagnoses occurring in children ≤ 2 years old.
Shin, 2024**	Four pathologies: pneumothorax, consolidation, nodule, pleural effusion. Mixed.	To evaluate whether optimal operating points of adult-oriented AI software differ for paediatric chest radiographs and assess diagnostic performance	Standard 15% operating point validated in previous adult studies (not stated in this publication)	When using standard adult operating points (15%), the AI achieved high overall AUC values (0.973–0.996) across all lesion types in children, but showed critically poor sensitivity for pneumothorax detection in those under 2 years old (sensitivity 0.5). After optimising operating points specifically for paediatric populations (11% for pneumothorax, 14% for consolidation, 6% for pleural effusion), the AI maintained similar AUC values but showed only modest improvement in the most vulnerable population, with pneumothorax sensitivity in children ≤ 2 years improving minimally from 0.5 to 0.6. This study demonstrates that adult-trained AI systems may have their performance improved in children by using simple threshold optimisation; however, this may still not be sufficient improvement for some critical scenarios, like for young children with pneumothoraces.

Studies investigating the same AI tool and/or patient group are marked with asterisks

**Table 3:** Focus areas for AI tool and task types for included studies (*n* = 20 articles)

Author, year	Body part	Modality	AI Tool	Disease/task	Classification of AI task
Candemir, 2015	Chest	Radiography (Chest)	Model-based lung boundary detection algorithm developed by the U.S. National Library of Medicine for chest X-ray screening. The algorithm uses anatomical atlases with non-rigid registration for lung segmentation.	Lung boundary detection	Segmentation task—specifically, lung boundary detection/segmentation to identify lung regions in chest X-ray images as a preprocessing step for abnormality classification.
Alqahtani, 2017*	MSK	Radiography (Lateral thoracolumbar spine, T4-L4)	COMMERCIAL PRODUCT SpineAnalyzer™ (Optasia Medical)—a semi-automated software program using the 6-point technique for vertebral fracture identification	Vertebral fractures	Detection and classification—the software detects vertebral fractures and classifies them based on vertebral height ratios
Alqahtani, 2019*	MSK	Radiography (Lateral spine) and DEXA (Thoracolumbar spine)	COMMERCIAL PRODUCT Two AI tools were evaluated: -AVERT™ (Optasia Medical)—a 33-point semi-automated program -SpineAnalyzer™ (Optasia Medical)—a 6-point semi-automated program	Vertebral fractures	Detection and classification—both software programs detect vertebral fractures and classify them
Alqahtani, 2020*	MSK	Radiography (Lateral spine) and DEXA (Thoracolumbar spine)	COMMERCIAL PRODUCT AVERT™ (Optasia Medical)	Vertebral fractures	Segmentation and classification—the software automatically outlines vertebral bodies with 33 measurement points and classifies vertebral fractures based on height loss ratios
Bermudez, 2020	Neuro	MRI (Brain, T1-w, 3 T field strength)	SLANT (Spatially Localised Atlas Network Tiles)—Academic tool, not commercial.	Whole brain segmentation	Segmentation of the whole brain on MRI studies
Shin, 2022**	Chest	Radiography (Chest)	COMMERCIAL PRODUCT Lunit Insight for Chest Radiography, v3	Eight detectable lesions on radiography: nodules, consolidation, fibrosis, atelectasis, cardiomegaly, pleural effusion, pneumothorax, pneumoperitoneum.	Detection and classification—detecting and scoring the presence of eight different types of thoracic abnormalities with abnormality scores presented as percentages.
Hardie, 2023	Chest	CT (Chest)	Two systems were tested: - FlyerScan: Traditional handcrafted-feature-based CAD system - MONAI (Medical Open Network for Artificial Intelligence): Deep learning-based convolutional neural network system using 3D-detection RetinaNet with ResNet backbone	Pulmonary nodules	Detection—specifically 3D bounding box detection of lung nodules with associated probability scores.
Morcos, 2023	Chest	Radiography (Chest)	TorchXRayVision “all” model—an open-source AI algorithm trained on seven different publicly available datasets	Pneumonia	Classification—distinguishing between healthy cases and pneumonia cases (with further subdivision into bacterial vs viral pneumonia).
Rajaraman, 2023	Chest	Radiography (Chest)	Multiple lung segmentation models were used, including UNet, TransUNet, and UNet variants with ImageNet-pretrained encoder backbones (VGG-16, VGG-19, Inception-V3, ResNet-50, DenseNet-121, EfficientNet-B0)	Lung boundary detection	Segmentation of the lung region
Salman, 2023***	Chest	CT (Chest and TAP)	COMMERCIAL PRODUCT Syngo.CT Lung CAD (VD20) (Siemens Healthineers)	Pulmonary nodules	Detection—identifying and marking pulmonary nodules.
Salman, 2023***	Chest	CT (Chest and TAP)	COMMERCIAL PRODUCT Syngo.CT Lung CAD (VD20) (Siemens Healthineers)	Pulmonary nodules	Detection—identifying and marking pulmonary nodules.
Yang, 2023	Thyroid	Ultrasound (Thyroid)	Deep learning algorithm developed by Duke University (DCNN-based)	Thyroid nodules	Classification—the model determines the likelihood of malignancy and risk stratification (categorises nodules into risk levels DL2 through DL5 with corresponding management recommendations).
Chatterjee, 2024	Whole Body	CT (Abdo/Pelvis)	TotalSegmentator (v1) by Nora Imaging Platform & RaySearch Labs (developed with nnU-Net architecture)	Organ segmentation (not disease specific)	Object segmentation—automated multi-organ segmentation of CT images
Chen, 2024	Head and Neck	CT (Head and Neck)	COMMERCIAL PRODUCT RSL RayMachine deep learning algorithm (v2.0.0.45) for auto-contouring head and neck structures integrated in RayStation v11B. Developed by RaySearch Laboratories AB.	Head and neck, and central nervous system tumours	Segmentation—automated contouring of sixteen organs at risk (OARs) in the head and neck region.
Kumar, 2024	Whole Body	CT (Chest/Abdomen)	nnU-Net framework—a self-configuring U-Net architecture for medical image segmentation.	Organ segmentation (not disease specific)	Segmentation—anatomical structures/organs in the chest and abdomen
Rollan-Martinez-Herrera, 2024	Chest	Radiography (Chest)	Custom pneumonia classification CNN with Xception backbone architecture. Developed by the research team using TensorFlow 2.15 with Xception as the backbone network.	Pneumonia	Classification—distinguishing between healthy cases and pneumonia cases (with further subdivision into bacterial vs viral pneumonia).
Lhermitte, 2024	Neuro	MRI (Brain, T1-w)	U-Net, Pretrained Res-U-Net, nnU-Net models	Stroke lesions	Segmentation of stroke lesions on the MRI brain
Shin, 2024**	Chest	Radiography (Chest)	COMMERCIAL PRODUCT Lunit Insight for Chest Radiography, v3.1.2	Four pathologies: pneumothorax, consolidation, nodule, pleural effusion	Detection and classification—detecting and scoring the presence of thoracic abnormalities
Ha, 2025	Thyroid	Ultrasound (Thyroid)	COMMERCIAL PRODUCT AI-Thyroid software, a Deep Learning model	thyroid nodules	Classification—identification of malignant thyroid nodules
Thibodeau-Antonacci, 2025	Whole body (organs at risk)	CT	COMMERCIAL PRODUCT LimbusAI v1.7 deep learning-based segmentation tool developed specifically for radiotherapy purposes	The Organs at risk (OARs) included: the clinical target volume of the spinal canal, vertebral bodies, brain, brainstem, eyes, lenses, optic nerves, chiasm, cochleae, oesophagus, lungs, and kidneys.	Segmentation—contouring of organs at risk (OARs) of paediatric patients in the context of paediatric craniospinal irradiation (CSI).

Studies investigating the same AI tool and/or patient group are marked with asterisks

*MSK* musculoskeletal, *CXR* chest radiograph, *DEXA* dual-energy X-ray absorptiometry, *CT TAP* CT thorax, abdomen and pelvis

### Study demographics

These studies collectively evaluated adult-trained AI models across different paediatric cohorts, with test datasets ranging from 30 to 7357 Paediatric subjects (Table 1). Most studies focused on school-aged children and adolescents. These studies represented work from diverse geographic regions: USA (*n* = 11) [19–23, 28, 29, 32, 35, 37, 38], UK (*n* = 3) [24, 25, 36], South Korea (*n* = 3) [26, 27, 37], Canada (*n* = 2) [30, 38] and one each from Australia [31], Austria [34], and France [33].

Three paediatric datasets were used by more than one study, although they were evaluating different AI model performances. Therefore, across all 20 publications, 16 distinct paediatric datasets were used. All were retrospective datasets, with the majority evaluating data from single-centre cohorts (8/16, 50.0%) [19–28, 34, 36] and 7 datasets (43.7%) [29, 31–33, 35, 37, 38] using multicentre datasets, predominantly open-source publicly available datasets. In one study, the origin of the dataset was not clearly disclosed [30]. Gender distribution within the paediatric datasets was not reported in 6 studies (37.5%) [19, 20, 30, 32, 35, 38], and was described in an unclear manner in 2 studies [31, 34] that quoted overall distribution from the wider dataset from which any paediatric subgroup data was derived. A commercially available AI tool was evaluated in 10 studies (7 individual products across these studies) [22–27, 34, 36–38], the remainder being research prototype/open-source models.

### Imaging modalities

Across the 16 datasets mentioned in the 20 publications, the two most common types of imaging modality evaluated in our review was radiography (6/16, 37.5%), predominantly chest radiographs (*n* = 5) [19, 20, 26, 27, 32, 35] and lateral thoracolumbar spine radiographs (*n* = 1) [36], together with CT (6/16, 37.5%) focussing on the chest (*n* = 2) [22, 23, 28], head and neck (*n* = 1) [34], chest and abdomen (*n* = 1) [31] abdomen/pelvis (*n* = 1) [29] and whole body (*n* = 1) [38]. Two studies evaluated MRI brain examinations [30, 33], one evaluated lateral thoracolumbar spine radiographs with dual-energy X-ray absorptiometry (DEXA) examinations [24, 25]. Only two studies evaluated ultrasound examinations (specifically thyroid ultrasound) [21, 37].

### AI-based tasks

AI tools were used for different tasks across these imaging modalities, which determined the diseases they were intended to assist in diagnosing or detecting (Table 2). Overall, 9 articles (45.0%) [25, 29–35, 38] evaluated AI tools for segmentation tasks (lung boundary segmentation, *n* = 2; multi-organ segmentation, *n* = 3; brain segmentation, *n* = 1; stroke lesions, *n* = 1; head and neck structures, *n* = 1; vertebral fractures, *n* = 1). Four articles (20.0%) [19–21, 37] reported the AI use as a classification tool (pneumonia, *n* = 2; thyroid nodules, *n* = 2), and 3 articles (15.0%) [22, 23, 28] used AI as a detection tool (all for pulmonary nodule detection). Four studies (22.2%) [24, 26, 27, 36] employed AI for multiple tasks involving both detection and classification (vertebral fractures, *n* = 2, lung pathologies, *n* = 2). Additional information regarding the focus of the AI tools and their tasks, as well as information on the study dataset size and demographics used for evaluating ‘adult-trained’ model performance, is presented (Tables 3 and 4).

**Table 4:** Study dataset size and demographics used for evaluating ‘adult-trained’ model performance (*n* = 20 articles)

Author, year	Modality	Dataset study period	Patient ages (years, unless otherwise stated)	Sample size	% Male in dataset	Number of centres the data derived from
Candemir, 2015	Radiography (Chest)	Reviewed data from ~2006–2017	Paediatric Group 1: 1 day ≤ Age < 2 years (52 CXRs) Paediatric Group 2: 2 years ≤ Age < 11 years (67 CXRs) Paediatric Group 3: 11 years ≤ Age < 18 years (42 CXRs) No mean, median, or standard deviation provided.	161 paediatric CXR images total, distributed across three age groups	*NS*	*Single*
Alqahtani, 2017*	Radiography (Lateral thoracolumbar spine, T4-L4)	November 2011–February 2014	5–15 years; mean 12 years.	137 subjects	33% (45 cases)	*Single*
Alqahtani, 2019*	Radiography (Lateral spine) and DEXA (Thoracolumbar spine)	November 2011–Feb 2014	5–15 years (mean 9.6 years)	50 subjects (100 lateral spine images: 50 DEXA + 50 radiographs)	42% (21/50)	*Single*
Alqahtani, 2020*	Radiography (Lateral spine) and DEXA (Thoracolumbar spine)	November 2011–November 2016	5–18 years (mean, median, s.d. not stated)	420 lateral DEXA scans (30 per year of age, 15 females and 15 males in each age group).	50% (210/420 cases)	*Single*
Bermudez, 2020	MRI (Brain, T1-w, 3 T field strength)	NS	2.34–4.31 years (no mean or median stated)	30 paediatric subjects (for retraining a paediatric model using transfer learning) 45 adult subjects (from the Open Access Series on Imaging Studies, OASIS; ages 18–96 years) for adult retraining transfer learning. For model development in this study, all were trained keeping 20% of the paediatric dataset as testing, with 80% for re-training/transfer learning	NS	*NS*
Shin, 2022**	Radiography (Chest)	March 2021–May 2021	< 18 years (mean 7.0 ± 5.8)	2273 paediatric chest radiographs	56.3% (1280/2273)	*Two centres, same city*
Hardie, 2023	CT (Chest)	Nov 30, 2018–Aug 31, 2020	4–17 years; mean 13.1 ± 3.3 years	59 patients (59 CT scans, one scan per patient), containing 1355 nodules (288 measuring 3–30 mm; 1067 micronodules measuring 1 mm to 3 mm)	51% (30/59)	*Single*
Morcos, 2023	Radiography (Chest)	Not specified as collected over a specific time period	Children from 1 to 5 years of age. Specific mean, median, s.d. not stated.	5856 total cases (1583 normal cases and 4273 pneumonia cases: 2780 bacterial pneumonia, 1493 viral pneumonia) The data was collected from an open-source dataset derived from the Guangzhou Women and Children’s Medical Center in Guangzhou, China.	*NS*	*Single*
Rajaraman, 2023	Radiography (Chest)	Time period for collection not stated.	Three paediatric groups (labelled P1, 2, 3 across different ages) were evaluated from wider dataset: P1 (1 day to < 24 months), P2 (24 months to < 11 years), P3 (11 years to < 18 years). Specific mean, median, s.d. not stated	The paediatric dataset was derived from a private collection of 161 frontal CXRs plus paediatric CXRs removed from adult datasets (Shenzhen, Montgomery, JSRT), giving 203 cases altogether. Various models were trained and tested on different aspects of this dataset. For evaluating the models trained entirely on adult data, the full 203 paediatric cases were used When a portion of the paediatric dataset was used to retrain the models, an independent subset of the wider data was used to measure performance. In this scenario, the paediatric test set comprised 11 (P1), 17 (P2) and 11 (P3) cases that were unseen and held out during the training and validation.	*NS*	*Single*
Salman, 2023***	CT (Chest and TAP)	Dec 20, 2021–Apr 12, 2022	12–18 years (mean 15.4 ± 2.1 years)	30 patients with CT chest examinations (derived from 22 CT chest examinations, and 8 CT chest, abdomen, pelvis examinations, 3 mm and 1 mm slice thickness, lung kernel)	47% (14/30)	*Single*
Salman, 2023***	CT (Chest and TAP)	Dec 20, 2021–Apr 12, 2022	12–18 years (mean 15.5 ± 2.1 years)	30 patients with CT chest examinations (derived from 22 CT chest examinations, and 8 CT chest, abdomen, pelvis examinations, 3 mm slice thickness, lung kernel)	47% (14 of 30)	*Multicentre*
Yang, 2023	Ultrasound (Thyroid)	Jan 1, 2004–Sep 18, 2020	≤ 21 years; median 17.5 years (IQR: 15.3–19.3 years)	139 patients with 139 thyroid ultrasound examinations (containing 83 benign, 56 malignant nodules; single nodule per ultrasound examination)	14.4% (20/139)	Single
Chatterjee, 2024	CT (Abdo/Pelvis)	Time period not specified—described as a publicly available dataset.	0–16 years (mean 6.9 ± 4.5)	359 paediatric CT chest/abdomen/pelvis studies overall for external validation derived from the paediatric Chest/Abdomen/Pelvic CT Exams with Expert Organ Contours (Paediatric-CT-SEG) dataset from The Cancer Imaging Archive (TCIA). When a dataset was required for independent testing of the models after adaptation methods, 18% of this dataset was held out (*n* = 64)	50% (180/359)	Multicentre
Chen, 2024	CT (Head and Neck)	Jan 2020–Sep 2022	Paediatric patients ranged from 1 to 17 years (the youngest patient was 1 year old, with an overall patient age range of 1–79 years, median 45 years). No specific mean, median, or standard deviation provided for the paediatric subset alone.	The model was tested on the author’s own dataset of 137 CT scans from 98 patients (of which 42 were paediatric CT scans from 26 children)	Overall study population (adult and children) were 48% male and 52% female. Not stated just for children.	Single
Kumar, 2024	CT (Chest/Abdomen)	Not specified as collected over a specific time period	Paediatric patients aged 0–16 years, divided into age groups: 0–2, 3–4, 5–6, 7–9, and 10–16 years. Specific mean, median, s.d. not stated.	In this study, the authors evaluated an adult-trained model when applied to paediatric CT images, but also a model trained only using paediatric datasets, and a third model using both paediatric and adult datasets. A broad paediatric dataset was curated from multiple sources: 359 CT datasets from The Cancer Imaging Archive (TCIA) Paediatric-CT-SEG data and 100 paediatric datasets from Peter MacCallum Cancer Centre clinical data. An independent dataset of data, a subgroup of the data above (30 adults and 50 paediatric) was used to evaluate the three models.	For the specific test dataset, this is not explicitly stated. In the wider paediatric dataset from which the test data is derived (i.e., TCIA) there were 180 males and 179 females.	Multi-source from various public and local imaging databases
Rollan-Martinez-Herrera, 2024	Radiography (Chest)	Not stated	1–5 years (mean, median, range not stated)	5856 paediatric chest X-ray images. The data was collected from an open-source dataset derived from the Guangzhou Women and Children’s Medical Center in Guangzhou, China.	Not stated	Single
Lhermitte, 2024	MRI (Brain, T1-w)	Not stated	1–4 years (mean age 37.15 months ± 12.9 months)	40 MRI examinations (from 20 children; 20 pre- and 20 post-therapy sessions for cerebral palsy motor therapy)	40% (8/20)	Multicentre
Shin, 2024**	Radiography (Chest)	March 2021–November 2021	< 18 years (mean 7.2 ± 6.1 for test set; 5.9 ± 6.0 for ‘exploring set’)	7357 (4727 test set + 2630 exploring set)	55% (test set: 2602/4727), 53% (exploring set: 1396/2630)	Single
Ha, 2025	Ultrasound (Thyroid)	January 2013–December 2022	mean age 15.5 ± 2.4 years	two paediatric cohorts at two institutions (*n* = 128; 103 girls)	19.5% (25 boys)	multicentre
Thibodeau-Antonacci, 2025	CT (Whole body, organs at risk)	February 2009 -October 2021	Dataset 1: 14.9 [10.3–17.3] years Dataset 2: 8.5 [6.0–11.0]	Dataset 1: CT scans from 27 paediatric patients undergoing craniospinal irradiation (CSI) were obtained from St. Jude Children’s Research Hospital, USA. Dataset 2: 16 CT scans were collected from the McGill University Health Centre, Canada	Not stated	multicentre

The dataset study period denotes the time frame over which the paediatric test dataset was acquired, unless explicitly stated otherwise. All datasets were retrospective in nature

Studies investigating the same AI tool and/or patient group are marked with asterisks

*CXR* chest radiograph, *NS* not stated, *DEXA* dual-energy X-ray absorptiometry, *CT* computed tomography

### Segmentation tasks

Most adult-trained segmentation models demonstrated significant performance degradation when applied to paediatric populations (Table 2). The most pronounced deterioration occurred in the youngest age groups. Candemir et al [35] found lung boundary segmentation accuracy dropped from 95.4% (adults) to 36.8% (children < 2 years), though optimisation techniques improved paediatric performance to 86%. Similarly, Kumar et al [31] reported adult organ segmentation models achieved Dice Similarity Coefficients (DSC) of 0.80–0.92 in adults but only 0.07–0.65 in children aged 0–2 years, with performance gradually improving in older paediatric subgroups (0.78–0.97 DSC when retrained with paediatric data). Whole-body organ segmentation studies consistently showed age-dependent performance patterns. Chatterjee et al [29] demonstrated that TotalSegmentator, an adult-trained CT organ segmentation tool, achieved a mean DSC of 0.81 in adults versus 0.73 in children (*p* < 0.001), with the worst performance drops observed in adrenal glands (40–49% reduction) and duodenum (30% reduction). The youngest children (0–4 years) showed the greatest performance degradation, particularly for anatomically proportionally different organs. Similar findings were reported in a study by Thibodeau-Antonacci et al [38], demonstrating a drop in performance, particularly for structures prone to age-related differences, including the spinal cord and the oesophagus. The authors also noted that age-related changes in the fat composition around internal organs such as the kidneys can affect their detection by auto-segmentation models.

Brain segmentation presented unique challenges, with Bermudez et al [30] showing the adult-trained SLANT algorithm achieved DSC 0.82 in paediatric populations compared to established adult performance benchmarks of 0.715–0.760. Stroke lesion segmentation models performed poorly in paediatric patients, with DSC scores of 0.40–0.57 compared to adult performance of 0.54–0.79 [33].

### Classification tasks

Adult-trained classification models showed variable but generally reduced performance in paediatric applications (Table 2). Pneumonia classification models demonstrated moderate transferability, with Morcos et al [20] reporting 76.5% accuracy in children compared to adult AUC scores of 0.7–1.0, representing performance at the lower end of the adult range. However, Rollan-Martinez-Herrera et al [19] found their adult-trained CNN maintained “reasonable performance” with AUC 0.82 in children versus 0.95 in adults (although still a reduction in absolute performance metrics). Thyroid nodule classification presented particular challenges, with Yang et al [21] demonstrating that while the adult-trained model maintained similar sensitivity (87.5% vs 87% in adults), specificity dropped substantially (36.1% vs 52%), suggesting increased false-positive rates in paediatric populations. In a recent study, Ha et al reported that the area under the receiver operating characteristic (AUROC) values in the paediatric cohort were close to those reported in adult cohorts, around 0.92. They also reported sensitivity and specificity values of 78.7–89.4% and 79.8–91.7% in the paediatric cohort.

### Detection tasks

Detection tasks showed the most severe performance degradation (Table 2). Pulmonary nodule detection systems performed poorly across multiple studies. Hardie et al [28] reported that traditional CAD sensitivity dropped from 83.9% (adults) to 68.4% (children), while deep learning approaches declined from 95.5% to 53.1%. Commercial lung CAD systems showed even worse performance, with Salman et al [22] reporting sensitivities of only 26–39% in adolescents aged 12–18 years, compared to the expected adult performance of 68–100%.

### Mixed tasks

Multi-organ detection and classification systems demonstrated age-dependent performance variations (Table 2). Shin et al [26] found that commercial chest radiography AI achieved 97–99% accuracy in adults but only 87.5% overall accuracy in children, with 81.5% of errors occurring in children ≤ 2 years old. After age-specific optimisation (i.e., operating point adaptation), performance in the same tool improved modestly but remained suboptimal for critical conditions like pneumothorax in very young children (sensitivity from 50% to 60%) [27].

### Age-related performance patterns

A consistent finding across all task types was the inverse relationship between patient age and AI performance degradation. Children under 2 years consistently showed the greatest performance drops, with gradual improvement in older paediatric age groups approaching adult-like anatomy [26, 27, 31, 32, 35].

### Adaptation strategies

Several studies explored methods to improve paediatric performance. Transfer learning approaches showed promise, with Bermudez et al [30]. demonstrating that augmented transfer learning (combining adult and paediatric training data) improved paediatric brain segmentation performance, while also preserving adult accuracy. Chatterjee et al [29] found that models trained exclusively on paediatric data or fine-tuned using paediatric datasets achieved Dice score improvements of between 47–79% (compared to the performance using adult-only models) for abdominal organ segmentation tasks. Performance metrics could also be improved for commercial AI tools (where it is not possible to retrain the model). Shin et al [27] found that adjusting the operating point for a model that could classify lung findings on chest radiographs, there was a mild improvement in sensitivity (from 50% to 60%) for pneumothorax detection in children < 2 years old. The authors also discovered that overall model performance could be increased from 87.5% to 96.9% if cardiomegaly was disregarded and the tool was not used in those < 2 years old [26].

## Discussion

This study reveals that adult-trained AI models consistently demonstrate reduced performance when applied to paediatric populations without adaptation, with the greatest performance deficits occurring in the youngest children (especially those < 2 years of age). Detection tasks appeared to show the most severe deterioration, while classification tasks showed more modest decreases.

The observed performance gaps likely reflect anatomical and pathophysiological differences between paediatric and adult populations. Detection tasks exemplified this challenge—pulmonary nodule detection required not only recognition of the nodule characteristics, but also an understanding of the background anatomical context. paediatric chest anatomy differs from adults, featuring a more barrel-shaped configuration, frequent motion artefacts from breathing during imaging, reduced lung volumes, and background airspace opacification due to poor compliance with breath-holding instructions. Similarly, lung segmentation models trained on adult chest radiographs demonstrated poor performance, struggling to accurately delineate the triangular lung configuration, elevated diaphragmatic positioning, and proportionally enlarged cardiac silhouette characteristic of infants. Vertebral fracture detection algorithms frequently misinterpret physiological vertebral wedging, a normal developmental variant in growing children, as pathological fractures. These anatomical variations collectively confound AI models trained exclusively on adult morphology. In addition, the drop in model performance may also arise when models are applied to external datasets, and decreased algorithm performance when applied to an external dataset has been shown to occur in the vast majority of models [39].

Surprisingly, not all AI applications demonstrated performance degradation. One study using AI for head and neck anatomy segmentation [34] showed equivalent performance between adult and paediatric populations when standard head positioning on the scan was maintained, but failed in both populations where this was non-standard. One study using AI for the classification of malignant thyroid nodules showed similar results to those found when the AI tool was used in adults [37]. Also, another study [31] found that AI models originally intended for adults, but re-trained using a combination of adult and paediatric datasets, performed similarly to those that were only retrained on paediatric-specific data when applied to a paediatric population. This challenges the assumption that paediatric-specific training is always optimal alone and suggests that adult data variability may enhance the performance in children for certain situations. Beyond retraining AI models, other strategies in our study were also reported to help improve paediatric performance of various AI models, such as registration adaptation of segmentation models [35], threshold optimisation [27] and selective disease targeting [26] when using the AI tool. These findings also align with broader literature that supports the use of transfer learning, federated learning and domain adaptation as viable approaches for paediatric AI development, offering practical pathways for device improvement [40–42].

While we reviewed AI tools that conducted a variety of tasks, one notable missing task was that of triage, for which our search results did not yield any original publications. Nonetheless, this is important to flag given the issuance of a public warning statement by the American College of Radiologists in 2022 [4] regarding concerns from the use of adult-trained AI triage tools leading to down-triaging of important paediatric findings and delaying subsequent management. It is also important to state that due to this review focussing on medical imaging specifically, we did not address issues regarding AI tools that fall into the category of foundation models for healthcare documentation and clinic consultation. It is important to note that these also present additional unique paediatric challenges including consent and privacy issues for minors, whether the tools understand when to highlight age-appropriate milestones, errors in dosing or are able to identify constellation of signs to flag rare paediatric conditions. These gaps could lead to inappropriate recommendations for children and would require appropriate governance and evaluation prior to clinical use [43].

Moving forward, several initiatives warrant priority. At the model development stage, paediatric imaging and healthcare datasets should follow the ACCEPT-AI guidelines for use of paediatric data in AI [44], greater availability of open-source paediatric datasets [45–47] could promote more dedicated model development, and access to federated learning platforms could accelerate model generalisability [48–50]. Computational advances to maximise the utility of small, heterogeneous datasets are also crucial [51].

At the pre-implementation stage, the paediatric community must continue to advocate for either dedicated paediatric tool development or rigorous adult model validation before clinical implementation. Robust governance frameworks must define when, how and whether adult AI tools can be safely used “off-label” in children, specifying required validation levels and appropriate circumstances. Post-implementation, AI literacy and training programs must educate healthcare staff to recognise when AI tools not designed for paediatric use may be unreliable [52]. A recent review confirmed that most regulated radiology AI medical devices have missing or unclear information regarding the appropriate use in children, with only four (4/213, 2%) of these clearly labelled for paediatric use [6]. Given that many AI tools marketed for paediatric use lack supporting performance evidence in children [5], urgent needs for clearer labelling and validation standards—such as the model card (a type of ‘nutrition label’ for AI tools) can help improve transparency [53]. Establishing AI registries where institutions share internal paediatric validation data would further help facilitate this knowledge sharing and improve system-wide safety [54–56].

### Strengths and limitations

This study acknowledges several limitations. Despite our rigorous efforts to conduct a comprehensive literature search, it is conceivable that additional studies may emerge subsequent to this. To mitigate this, we revisited and updated our search strategy immediately before submitting the article. It is also important to note that our methodology did not extend to directly engaging with vendors or regulatory bodies to review external validation data of algorithms claiming to be suitable for paediatric populations without published articles demonstrating performance in children. There is a possibility that relevant studies, potentially yielding unfavourable results, remain undisclosed. Second, dataset heterogeneity in this review precluded formal meta-analysis, yet the consistent pattern of performance degradation across diverse applications and modalities provides compelling evidence of a repeated, rather than isolated, concern for use of ‘adult models’ in children. Furthermore, a few of the included papers in this scoping review report on the same AI tool or same patient group, but with different aims for each publication (Table 2). Finally, all included studies were retrospective, which limits the real-world applicability of the actual harms that may emerge in clinical practice. Nonetheless, this approach is a vital first step to predict possible safety risks before clinical deployment. In situations where some models appear useful and accurate for paediatric use on retrospective datasets, cautious prospective validation with appropriate guardrails will be essential to determine real-world utility.

## Conclusion

The findings emphasise that AI models designed for adults are unlikely to perform equivalently in paediatric populations, highlighting the critical need for paediatric-specific AI model development and validation. Healthcare professionals should not assume that adult-trained AI models can be directly generalised to paediatrics patients without fine-tuning with paediatric data. While emerging optimisation approaches show promise for bridging this performance gap, significant barriers including limited paediatric datasets, cohort overlap in the external validation, regulatory complexities and reduced commercial incentives must also be addressed to advance paediatrics AI tools for radiology safely and effectively.

## Supplementary information

Supplementary information

## Abbreviations

AI: Artificial intelligence
AI-SaMD: Artificial intelligence-based software as a medical device
AUC: Area under the curve
AUROC: Area under the receiver operating characteristic
CAD: Computer-aided detection
CNN: Convolutional neural network
DEXA: Dual-energy x-ray absorptiometry
DSC: Dice similarity coefficient
FDA: Food and Drug Administration
OSF: Open science framework
