# Supplementary material for: Performance of adult-trained artificial intelligence models in paediatric imaging—a scoping review
Source: Eur Radiol. 2026 Feb 12;36(7):5916–39. doi: 10.1007/s00330-026-12354-5 (PMC13282355; doi:10.1007/s00330-026-12354-5)
Supplement: Supplementary file 1 — Supplementary information [file 330_2026_12354_MOESM1_ESM.pdf]

## Performance of Adult-Trained Artificial Intelligence Models in Paediatric Imaging - A Scoping Review

## ELECTRONIC SUPPLEMENTARY MATERIAL

**Supplementary Table 1: Risk of bias and applicability of articles evaluated in this scoping review (n = 20).**

| Author, year | Risk of Bias | | | Applicability Concerns | | |
|---|---|---|---|---|---|---|
| | Patient Selection | Index Test (AI tool) | Reference Standard | Patient Selection | Index Test (AI tool) | Reference Standard |
| ***Candemir, 2015*** | **Low** – wide range of paediatric patients across different developmental stages. Single centre. | **High** – adult model | **Unclear** – manual delineations mentioned but level of readers not specified | **Low** – single source, but broad range of patient ages represented. | **High** | **Low** – manual lung boundary delineation acceptable standard |
| ***Alqahtani, 2017*** | **High** – majority children had reduced bone mineral density in association with rare diseases. | **High** – adult AI tool | **Low** – consensus of 3 paediatric radiologists. | **High** – enriched disease cohort. | **High** | **Low** – accepted reference. |
| ***Alqahtani, 2019*** | **High** – majority children had reduced bone mineral density in association with rare diseases. | **High** – adult AI tool | **Low** – consensus of 3 paediatric radiologists. | **High** – enriched speciality cohort. | **High** | **Low** – appropriate reference. |
| ***Alqahtani, 2020*** | **High** – many had rare bone diseases | **High** – adult tool | **Low** – consensus of 3 paediatric radiologists. | **High** – rare-disease heavy cohort, not general paediatrics. | **High** | **Low** – appropriate reference. |
| ***Bermudez, 2020*** | **Unclear** – small dataset of MRI studies, without clear exclusion criteria. | **High** – adult AI tool | **Low** – expert manual correction and segmentation. | **High** – narrow age and research context. | **High** | **Low** – expert-corrected masks appropriate. |

| Author, year | Risk of Bias | | | Applicability Concerns | | |
|---|---|---|---|---|---|---|
| | Patient Selection | Index Test (AI tool) | Reference Standard | Patient Selection | Index Test (AI tool) | Reference Standard |
| ***Shin, 2022*** | **Low** – included all patient ages and clear exclusions specified. | **High** – adult commercial AI | **Low** – single paediatric radiologist, with standard clinical information. | **High** – single-region practice; age mix skews issues ≤2 yrs. | **High** | **Low** – experienced single-reader reference common in practice. |
| ***Hardie, 2023*** | **Low** – wide range of nodule sizes and clear exclusion criteria stated | **High** – adult-trained CAD | **Low** – multi-reader paediatric radiologist workflow for nodule detection and annotation. | **High** – oncology only. | **High** | **Low** – expert annotations appropriate. |
| ***Morcos, 2023*** | **Low** – Included a large dataset of radiographs for a selective age group of children (ages 1–5) | **High** – TorchXRayVision trained mainly on adults (minor paeds <5%). | **Low** – labels by two expert physicians, although unclear if they were blinded or not. | **High** – single centre/age band. | **High** | **Low** – pragmatic dataset standard. |
| ***Rajaraman, 2023*** | **Low** – paediatric subsets defined across stages, but small datasets. | **High** – adult segmentation models cross-domain; limited external paeds. | **Unclear** – paediatric ground truth labelling not explicitly described. | **High** – small dataset. | **High** | **High** – ground truthing process not fully specified. |
| ***Salman, 2023 (Clin Imaging)*** | **Low** – 30 consecutive, 12–18 yrs; clear exclusions. | **High** – adult CAD | **Low** – two paediatric radiologists, blinded, consensus. | **High** – older teens only, single institution. | **High** | **Low** – expert consensus appropriate |
| ***Salman, 2023 (Eur J Pediatr)*** | **Low** – same cohort as prior study, clear exclusions listed. | **High** – adult CAD | **Low** – two paediatric radiologists, blinded, consensus. | **High** – only teenagers, small single centre cohort. | **High** | **Low** – consensus standard. |
| ***Yang, 2023*** | **High** – only pathology-confirmed nodules so biased selection | **High** – AI trained on adults. | **Low** – histopathology is a good reference for | **High** – older teens dominate; tertiary. | **High** | **Low** – appropriate standard for evaluating |

| Author, year | Risk of Bias | | | Applicability Concerns | | |
|---|---|---|---|---|---|---|
| | Patient Selection | Index Test (AI tool) | Reference Standard | Patient Selection | Index Test (AI tool) | Reference Standard |
| | | | defining malignancy | | | presence of malignancy |
| ***Chatterjee, 2024*** | **Low** – reasonable dataset across multiple bony parts for review. | **High** – adult TotalSegmentator baseline; paeds models only after fine-tuning/training. | **Low** – expert contours reviewed by radiation oncologist | **Low** – broad paediatric spectrum of body parts reviewed across different CT indications. | **High** | **Low** – accepted standard. |
| ***Chen, 2024*** | **Low** – reasonable dataset in variety of positions and immobilisation devices. | **High** – adult commercial model | **Low** – manual contours by radiation therapy team. | **High** – small paediatrics subset | **High** | **Low** – accepted clinical practice. |
| ***Kumar, 2024*** | **Low** – multi-source data with clear age stratifications. | **High** – adult-only model | **Low** – expert contours | **Low** – broad age range & scanners | **High** | **Low** – appropriate reference |
| ***Rollan-Martinez-Herrera, 2024*** | **Low** – Large paediatric public dataset across different diseases, clear exclusion criteria. | **High** – adult-trained model. | **Low** – labels by three physicians; although no microbiologic gold standard. | **High** – 1–5 yrs, single centre dataset. | **High** | **Low** – radiographic labels acceptable. |
| ***Lhermitte, 2024*** | **Unclear** – Small dataset but across multiple sites. | **High** – adult segmenters (U-Net/Res-U-Net/nnU-Net) transferred; tiny paeds sample. | **Unclear** – medical experts mentioned for segmentation checking, but not specified how experienced or trained. | **High** – CP/post-stroke subgroup. | **High** | **Low** – accepted segmentation approach. |
| ***Shin, 2024*** | **Low** – included all patient ages and clear exclusions specified. | **High** – adult commercial AI | **Low** – single paediatric radiologist, with standard clinical information. | **High** – single-region practice. | **High** | **Low** – experienced single-reader reference common in practice. |

| Author, year | Risk of Bias | | | Applicability Concerns | | |
|---|---|---|---|---|---|---|
| | Patient Selection | Index Test (AI tool) | Reference Standard | Patient Selection | Index Test (AI tool) | Reference Standard |
| ***Ha, 2025*** | **High** – very selective cases of nodules above a certain size, undergoing both imaging and FNA. | **High** – adult-trained AI-Thyroid model | **Low** – histopathology gold standard is acceptable for evaluating malignancy. | **High** – Older teens dominant; FNA-selected. | **High** | **Low** – appropriate reference for malignancy |
| ***Thibodeau-Antonacci, 2025*** | **Low** – small cohort of cases from one centre, although included a wide range of organs at risk (OAR) for segmentation per study. | **Unclear** – mixed index tests (commercial adult LimbusAI + nnU-Net/in-house); external test present but heterogeneous. | **Unclear** – all contours manually delineated/curated; clinical acceptability scored but no mention of who did this. | **High** – oncology paediatric cases, not for general paediatric cases. Small dataset evaluated. | **High** | **Unclear** – manual delineation of organs for segmentation is acceptable standard but not clear who conducted these. |

**Adult artificial intelligence models used in pediatric radiology**

**Ovid MEDLINE(R) and Epub Ahead of Print, In-Process, In-Data-Review & Other Non-Indexed Citations, Daily and Versions <1946 to June 23, 2025>; Search date 24 June 2025**

1 artificial intelligence/ or exp machine learning/ or deep learning/ 144359
2 neural networks, computer/ 62269
3 (artificial adj1 intelligence).ti,ab,kf. 77943
4 ((deep or machine) adj2 learning).ti,ab,kf. 223654
5 (AI or DL or DLS or CNN or DCNN).ti,ab,kf. 279911
6 ((deep or convolutional or neural) adj3 network*).ti,ab,kf. 142164
7 1 or 2 or 3 or 4 or 5 or 6 600487
8 exp Radiology/ 46026
9 diagnostic imaging/ or exp image interpretation, computer-assisted/ or exp radiography/ or exp tomography, emission-computed/ or exp tomography/ or exp ultrasonography/ 2266328
10 diagnostic imaging.fs. 1575419
11 (imag* or radiolog* or radiogra* or ultraso* or sonogra* or tomogra*).ti,ab,kf. 2850555
12 8 or 9 or 10 or 11 4021432
13 7 and 12 151815
14 exp Pediatrics/ 65398
15 adolescent/ or exp child/ or exp infant/ or young adult/ 4686226
16 (pediatr* or paediatr* or child* or neonat* or infan* or baby or babies or fetal or adolescen* or newborn* or new-born* or perinat* or toddler* or minors* or boy or boys or boyhood or girl* or kid or kids or schoolchild* or school child* or juvenil* or youth* or teen* or under*age* or pubescen* or young adult*).ti,ab,kf. 3489502
17 14 or 15 or 16 5901304
18 7 and 12 and 17 14854
19 Adult/ or exp aged/ or middle aged/ 8321236
20 (adult* or man or men or woman or women).ti,ab,kf. 3710367
21 19 or 20 9951656
22 7 and 12 and 17 and 21 8058
23 (validation or validated or performance).ti,ab,kf. 2200116
24 22 and 23 2501
25 limit 24 to yr="2014 -Current" 2214

https://ovidsp.ovid.com/ovidweb.cgi?T=JS&NEWS=N&PAGE=main&SHAREDSEARCHID=2SIoH6PARvjzIBQVhcteX940LMMhclmAjftQyS5efo13Vvkj2xfrISV4gctk5zHtt

Comments: for OVID-databases
/ = search on subject heading
Exp = search on a subject heading, expanded to include narrower terms
Ti,ab,kf = search in title, abstract and authors keywords of the article
Adjn = search terms from to each other. The adjacency operator (**ADJn**) retrieves records that contain search terms within a specified number (*n-1*) of words from each other in any order

**Embase (Ovid) <1974 to 2025 June 23>; Search date 24 June 2025**

1 artificial intelligence/ or deep learning/ or machine learning/ or supervised machine learning/ or unsupervised machine learning/ or artificial neural network/ or convolutional neural network/ or deep neural network/ 368899
2 (artificial adj1 intelligence).ti,ab,kf. 90474
3 ((deep or machine) adj2 learning).ti,ab,kf. 257206
4 (AI or DL or DLS or CNN or DCNN).ti,ab,kf. 486060
5 ((deep or convolutional or neural) adj3 network*).ti,ab,kf. 165457
6 1 or 2 or 3 or 4 or 5 875888
7 radiology/ 62290
8 diagnostic imaging/ 276649
9 exp radiography/ 1451346
10 exp tomography/ or exp computer assisted tomography/ or exp nuclear magnetic resonance imaging/ or exp x-ray tomography/ or exp echography/ 3613832
11 (imag* or radiolog* or radiogra* or ultraso* or sonogra* or tomogra*).ti,ab,kf. 3928319
12 7 or 8 or 9 or 10 or 11 6011548
13 6 and 12 245627
14 exp pediatrics/ 135047
15 exp infant/ or exp child/ or newborn/ 3423964
16 exp adolescent/ or young adult/ 2396695
17 (pediatr* or paediatr* or child* or neonat* or infan* or baby or babies or fetal or adolescen* or newborn* or new-born* or perinat* or toddler* or minors* or boy or boys or boyhood or girl* or kid or kids or schoolchild* or school child* or juvenil* or youth* or teen* or under*age* or pubescen* or young adult*).ti,ab,kf. 4410852
18 14 or 15 or 16 or 17 6220938
19 6 and 12 and 18 27207
20 adult/ or exp aged/ or middle aged/ 12431680
21 (adult* or man or men or woman or women).ti,ab,kf. 5117312
22 20 or 21 14146033
23 6 and 12 and 18 and 22 14180
24 (validation or validated or performance).ti,ab,kf. 2780725
25 23 and 24 3383
26 limit 25 to yr="2014 -Current" 3164
27 limit 26 to conference abstract 573
28 26 not 27 2591

Comments
line 7: radiology: not expanded in this database due to zero narrower terms
Note: “diagnostic imaging” not existing as subheading in EMBASE

https://ovidsp.ovid.com/ovidweb.cgi?T=JS&NEWS=N&PAGE=main&SHAREDSEARCHID=4YwMucfcawrftaY8jB5EK6pVehM6f8buCf3qopn91v4QDnEDosp10CjTnVsvbLJLR

**Cochrane Library (Wiley; Search date 24 June 2025**

#1 (artificial NEAR/1 intelligence):ti,ab,kw 2930
#2 ((deep or machine) NEAR/2 learning):ti,ab,kw 4382

#3 ((AI or DL or DLS or CNN or DCNN)):ti,ab,kw 30115
#4 ((deep or convolutional or neural) NEAR/3 network*):ti,ab,kw 2198
#5 #1 or #2 or #3 or #4 36023
#6 (imag* or radiolog* or radiogra* or ultraso* or sonogra* or tomogra*):ti,ab,kw 225309
#7 #5 and #6 5957
#8 (pediatr* or paediatr* or child* or neonat* or infan* or baby or babies or fetal or adolescen* or newborn* or new-born* or perinat* or toddler* or minors* or boy or boys or boyhood or girl* or kid or kids or schoolchild* or (school NEXT child*) or juvenil* or youth* or teen* or under*age* or pubescen* or (young NEXT adult*)):ti,ab,kw 466060
#9 (adult* or man or men or woman or women):ti,ab,kw 1026727
#10 #5 and #6 and #8 and #9 519
#11 (validation or validated or performance):ti,ab,kw 180558
#12#5 and #6 and #8 and #9 and #11 183

Limited to 2014-2025: **150** results (0 syst.rev, 150 trials)

Comments:
ti,ab,kw = search in title, abstract and subject headings
Near/2 = The adjacency operator (NEAR/**n**) retrieves records that contain search terms within a specified number (*n*) of words between the search terms in any order

**Web of Science (Clarivate) covering WOS.SCI: 1945 to 2024, WOS.AHCI: 1975 to 2024, WOS.ESCI: 2020 to 2025, WOS.SSCI: 1956 to 2024; >; Search date 24 June 2025**

1: TS=(artificial NEAR/0 intelligence) Results: 194934
2: TS=((deep or machine) NEAR/1 learning) Results: 610262
3: TS=(AI or DL or DLS or CNN or DCNN) Results: 491876
4: TS=((deep or convolutional or neural) NEAR/2 network*) Results: 585874
5: #4 OR #3 OR #2 OR #1 Results: 1394954
6: TS=(imag* or radiolog* or radiogra* or ultraso* or sonogra* or tomogra*) Results: 4382407
7: #5 AND #6 Results: 295647
8: TS=(pediatr* or paediatr* or child* or neonat* or infan* or baby or babies or fetal or adolescen* or newborn* or new-born* or perinat* or toddler* or minors* or boy or boys or boyhood or girl* or kid or kids or schoolchild* or "school child*" or juvenil* or youth* or teen* or under*age* or pubescen* or "young adult*")
Results: 4465402

9: TS=(adult* or man or men or woman or women) Results: 4791663
10: #5 AND #6 AND #8 AND #9 Results: 2207
11: TS=(validation or validated or performance) Results: 7080496
12: #5 AND #6 AND #8 AND #9 AND #11 Results: 739
13: #5 AND #6 AND #8 AND #9 AND #11 and 2014 or 2015 or 2016 or 2017 or 2018 or 2019 or 2020 or 2021 or 2022 or 2023 or 2024 or 2025 (Publication Years)
Results: 679

**https://www.webofscience.com/wos/woscc/summary/43975d42-b54c-4a7f-92e1-7ee7845128f9-016a88b460/relevance/1**

Comments:
TS = Topic search = search in title, abstract and author key words
Near/2 = The adjacency operator (NEAR/**n**) retrieves records that contain search terms within a specified number (*n*) of words between the search terms in any order

Search terms for AI were inspired by the systematic review:
Vandevenne MMS, Favuzza E, Veta M, Lucenteforte E, Berendschot TTJM, Mencucci R, Nuijts RMMA, Virgili G, Dickman MM. Artificial intelligence for detecting keratoconus. Cochrane Database of Systematic Reviews 2023, Issue 11. Art. No.: CD014911. DOI: 10.1002/14651858.CD014911.pub2.
https://www.cochranelibrary.com/cdsr/doi/10.1002/14651858.CD014911.pub2/appendices#CD014911-sec-0058

# Preferred Reporting Items for Systematic reviews and Meta-Analyses extension for Scoping Reviews (PRISMA-ScR) Checklist

| SECTION | ITEM | PRISMA-ScR CHECKLIST ITEM | REPORTED ON PAGE # |
|---|---|---|---|
| **TITLE** | | | |
| Title | 1 | Identify the report as a scoping review. | 1 |
| **ABSTRACT** | | | |
| Structured summary | 2 | Provide a structured summary that includes (as applicable): background, objectives, eligibility criteria, sources of evidence, charting methods, results, and conclusions that relate to the review questions and objectives. | 1 |
| **INTRODUCTION** | | | |
| Rationale | 3 | Describe the rationale for the review in the context of what is already known. Explain why the review questions/objectives lend themselves to a scoping review approach. | 3 |
| Objectives | 4 | Provide an explicit statement of the questions and objectives being addressed with reference to their key elements (e.g., population or participants, concepts, and context) or other relevant key elements used to conceptualize the review questions and/or objectives. | 3 |
| **METHODS** | | | |
| Protocol and registration | 5 | Indicate whether a review protocol exists; state if and where it can be accessed (e.g., a Web address); and if available, provide registration information, including the registration number. | 3 |
| Eligibility criteria | 6 | Specify characteristics of the sources of evidence used as eligibility criteria (e.g., years considered, language, and publication status), and provide a rationale. | 4 |
| Information sources* | 7 | Describe all information sources in the search (e.g., databases with dates of coverage and contact with authors to identify additional sources), as well as the date the most recent search was executed. | 4 |
| Search | 8 | Present the full electronic search strategy for at least 1 database, including any limits used, such that it could be repeated. | Suppl.material |
| Selection of sources of evidence† | 9 | State the process for selecting sources of evidence (i.e., screening and eligibility) included in the scoping review. | 4 |
| Data charting process‡ | 10 | Describe the methods of charting data from the included sources of evidence (e.g., calibrated forms or forms that have been tested by the team before their use, and whether data charting was done independently or in duplicate) and any processes for obtaining and confirming data from investigators. | 4 |
| Data items | 11 | List and define all variables for which data were sought and any assumptions and simplifications made. | 4 |
| Critical appraisal of individual sources of evidence§ | 12 | If done, provide a rationale for conducting a critical appraisal of included sources of evidence; describe the methods used and how this information was used in any data synthesis (if appropriate). | 4 |

| SECTION | ITEM | PRISMA-ScR CHECKLIST ITEM | REPORTED ON PAGE # |
|---|---|---|---|
| Synthesis of results | 13 | Describe the methods of handling and summarizing the data that were charted. | 4 |
| **RESULTS** | | | |
| Selection of sources of evidence | 14 | Give numbers of sources of evidence screened, assessed for eligibility, and included in the review, with reasons for exclusions at each stage, ideally using a flow diagram. | 5 |
| Characteristics of sources of evidence | 15 | For each source of evidence, present characteristics for which data were charted and provide the citations. | 5 |
| Critical appraisal within sources of evidence | 16 | If done, present data on critical appraisal of included sources of evidence (see item 12). | na |
| Results of individual sources of evidence | 17 | For each included source of evidence, present the relevant data that were charted that relate to the review questions and objectives. | 5 |
| Synthesis of results | 18 | Summarize and/or present the charting results as they relate to the review questions and objectives. | 5-8 |
| **DISCUSSION** | | | |
| Summary of evidence | 19 | Summarize the main results (including an overview of concepts, themes, and types of evidence available), link to the review questions and objectives, and consider the relevance to key groups. | 9 |
| Limitations | 20 | Discuss the limitations of the scoping review process. | 11 |
| Conclusions | 21 | Provide a general interpretation of the results with respect to the review questions and objectives, as well as potential implications and/or next steps. | 11 |
| **FUNDING** | | | |
| Funding | 22 | Describe sources of funding for the included sources of evidence, as well as sources of funding for the scoping review. Describe the role of the funders of the scoping review. | na |

JBI = Joanna Briggs Institute; PRISMA-ScR = Preferred Reporting Items for Systematic reviews and Meta-Analyses extension for Scoping Reviews.
* Where *sources of evidence* (see second footnote) are compiled from, such as bibliographic databases, social media platforms, and Web sites.
† A more inclusive/heterogeneous term used to account for the different types of evidence or data sources (e.g., quantitative and/or qualitative research, expert opinion, and policy documents) that may be eligible in a scoping review as opposed to only studies. This is not to be confused with *information sources* (see first footnote).
‡ The frameworks by Arksey and O'Malley (6) and Levac and colleagues (7) and the JBI guidance (4, 5) refer to the process of data extraction in a scoping review as data charting.
§ The process of systematically examining research evidence to assess its validity, results, and relevance before using it to inform a decision. This term is used for items 12 and 19 instead of "risk of bias" (which is more applicable to systematic reviews of interventions) to include and acknowledge the various sources of evidence that may be used in a scoping review (e.g., quantitative and/or qualitative research, expert opinion, and policy document).

*From:* Tricco AC, Lillie E, Zarin W, O'Brien KK, Colquhoun H, Levac D, et al. PRISMA Extension for Scoping Reviews (PRISMAScR): Checklist and Explanation. Ann Intern Med. 2018;169:467–473. doi: 10.7326/M18-0850.
